# Negative Regulation of CPSF6 Suppresses the Warburg Effect and Angiogenesis Leading to Tumor Progression Via c-Myc Signaling Network: Potential Therapeutic Target for Liver Cancer Therapy

**DOI:** 10.7150/ijbs.93462

**Published:** 2024-06-17

**Authors:** Deok Yong Sim, Hyo-Jung Lee, Chi-Hoon Ahn, JiEon Park, Su-Yeon Park, Bum-Ju Kil, Bum-Sang Shim, Bonglee Kim, Sung-Hoon Kim

**Affiliations:** Cancer Molecular Targeted Herbal Research Laboratory, College of Korean Medicine, Kyung Hee University, 26 Kyungheedae-ro, Dongdaemun-gu, Seoul 02447.

**Keywords:** CPSF6, Warburg effect, angiogenesis, hepatocellular carcinoma, c-Myc

## Abstract

In this study, we explored the oncogenic mechanism of cleavage and polyadenylation-specific factor 6 (CPSF6) in hepatocellular carcinoma (HCC). CPSF6 was overexpressed in HCC tissues with poor survival rates compared to normal tissues. Hence, CPSF6 depletion suppressed cell viability and colony formation, induced apoptosis via PARP cleavage, and increased the sub-G1 population of Hep3B and Huh7 cells. In addition, CPSF6 enhanced the stability of c-Myc via their binding through nuclear co-localization by binding to c-Myc at the site of 258-360. Furthermore, c-Myc degradation by CPSF6 depletion was disturbed by FBW7 depletion or treatment with the proteasomal inhibitor MG132. Additionally, CPSF6 depletion suppressed the Warburg effect by inhibiting glucose, HK2, PKM2, LDH, and lactate; showed a synergistic effect with Sorafenib in Hep3B cells; and inhibited angiogenesis by tube formation and CAM assays, along with decreased expression and production of vascular endothelial growth factor (VEGF). Notably, CPSF6 depletion attenuated PD-L1 expression and increased Granzyme B levels, along with an increase in the percentage of CD4/CD8 cells in the splenocytes of BALB/c nude mice bearing Hep3B cells. Consistently, immunohistochemistry showed that CPSF6 depletion reduced the growth of Hep3B cells in BALB/c mice in orthotopic and xenograft tumor models by inhibiting tumor microenvironment-associated proteins. Overall, these findings suggest that CPSF6 enhances the Warburg effect for immune escape and angiogenesis, leading to cancer progression via c-Myc, mediated by the HK, PD-L1, and VEGF networks, with synergistic potential with sorafenib as a molecular target for liver cancer therapy.

## Introduction

Hepatocellular carcinoma (HCC) accounts for approximately 90% of liver cancer 1 as the third leading cause of cancer-associated mortality worldwide 2. HCC progression is critically associated with signaling pathways related to metabolism 3, angiogenesis 4 and immune escape 5, as HCC is regarded as a genetically and phenotypically heterogeneous cancer within tumor microenvironment (TME) 6.

In the TME, dysregulated metabolism is regarded as a driving force for tumor initiation and progression 7, 8 because cancer cells prefer aerobic metabolism for oxidative phosphorylation, which is known as the Warburg effect 9. Thus, lactate production during cancer promotes malignant cancer progression through metastasis, angiogenesis, and immunosuppression, resulting in poor prognosis 10, 11. Additionally, extracellular acidification by the Warburg effect enhances immune evasion capacity of cancer cells via an increased ratio of M1-/M2-like macrophage populations 12, 13. Emerging evidence indicates that HCCs can evade immune surveillance without being recognized by antigen-presenting cells (APCs). Thus, PD-L1+ Kupffer cells interact with PD-1 + CD8+ T cells to suppress T cell immunity 14 and elevated PD-L1 expression is critically associated with poor prognosis in patients with HCC 15.

In addition, angiogenesis critically contributes to HCC progression via the VEGF/VEGF receptor signaling pathway 4, 16, which is targeted by tyrosine kinase inhibitors such as sorafenib 17.

The *Myc* family, consisting of *c-Myc*, *N-Myc*, *L-Myc*, *S-Myc*, and c-Myc oncogenes 18, 19 modulates genes involved in biogenesis and glucose and glutamine metabolism 20, 21, and is regulated by a variety of E3 ligases and the ubiquitin proteasome system (UPS), including the F-box protein FBW7 22.

Among the potent molecular target oncogenes, cleavage and polyadenylation-specific factor 6 (CPSF6) is a component of the cellular cleavage and polyadenylation complex 23. As a fusion partner of FGFR1 (fibroblast growth factor receptor 24, CPSF6 is involved in the progression of acute myeloid leukemia 25, HIV-1 infection 26, breast cancer 27, gastric cancer 28 and liver cancer 29. However, the molecular mechanisms underlying the effects of CPSF6 remain unclear. Thus, in the present study, the oncogenic mechanism of CPSF6 was elucidated in HCC tissues, cell lines, and orthotopic and xenograft tumor models in association with the Warburg effect, which is closely associated with angiogenesis and immune escape in the TME.

## Materials and Methods

### Cell lines and cell culture

Human HCC cell lines, HepG2, Hep3B, Huh7, and Sk-hep-1, and human embryonic kidney Hek-293T cells were purchased from the American Type Culture Collection (Manassas, VA, USA). HepG2, Hep3B, Sk-hep-1 and Hek-293T cells were cultured in Dulbecco's modified Eagle's medium (DMEM; Catalog No. LM 001-05, WelGENE, Republic of Korea). Huh7 cells were cultured in Roswell Park Memorial Institute 1640 medium (catalog no. LM 011-01, WelGENE, Republic of Korea). All the cells were cultured at 37°C in the aforementioned medium supplemented with 10% fatal bovine serum and 1% antibiotic solution (100 units/mL penicillin and 100 µg/mL streptomycin) in a 5% CO_2_ atmosphere. In addition, all cell lines were authenticated by short tandem repeat profiling without mycoplasma contamination.

### cDNA microarray measurement and data analysis

Total RNA was isolated from Hep3B cells transfected with the control vector and CPSF6 siRNA using TRIzol reagent (Invitrogen). The RNA quality was assessed using an Agilent 2100 bioanalyzer, including the RNA 6000 Nano Chip (Agilent Technologies, Amstelveen, Netherlands), and RNA quantification was conducted using an ND-2000 Spectrophotometer (Thermo Inc., DE, USA). Based on the library preparation and sequencing described by Kim *et al.* 30, data analysis was performed. Differentially expressed genes were determined based on the counts from unique and multiple alignments using Bedtools (Quinlan, 2010). The Read Count data were processed based on the TMM + CPM normalization method using EdgeR within R (R development Core Team, 2020) and Bioconductor (Gentleman *et al.*, 2004). Gene classification was based on searches conducted using the DAVID (https://david.ncifcrf.gov/) and Medline databases (https://www.ncbi.nlm.nih.gov/). The mRNA-sequencing data used in this study (GSE229281) are available from the NCBI public repository (https://www.ncbi.nlm.nih.gov/geo/query/acc.cgi?acc=GSE229281).

### RNA interference and plasmid transfection

HCC cells were seeded onto culture plate overnight and transfected with CPSF6 siRNA or FBW7 siRNA or SKP2 siRNA or cMyc siRNA or negative control siRNA purchased from Bioneer (Korea) adjusted at 40 nM by using transfection reagent (INTERFERin®, Polyplus, France) according to manufacturer's protocols. The transfected cells were incubated for 48 h for next experiment. In addition, CPSF6, V5-c-Myc, HA-ubiquitin, HA-c-Myc WT, HA-Fbw7, and pcDNA 3.0, plasmids were purchased from Addgene (Watertown, MA, USA), while HA-c-Myc three-domain plasmids were kindly provided by Prof. Hua Lu (Tulane University, New Orleans, USA) 31 for transfection into Hek-293T cells using transfection reagent (Turbofect, Thermo Fisher Scientific, Radnor, PA, USA).

### Cytotoxicity assay

Hep3B and Huh7 cells were transfected with CPSF6 or negative control siRNA for 48 h. The cells were seeded into 96 well plate at a density of 7 × 10 ^3^ cells/well and incubated overnight at 37 ℃ for 24, 48, and 72 h. Thereafter 20 μL of MTT (3-(4,5-dimethylthiazol-2-yl)-2,5-diphenyltetrazolium bromide, 1 mg/mL, 4 Merck KGaA, Germany) was added to each well of the plate and incubated for 2 h at 37 ℃ in dark. After the supernatant was carefully aspirated, 100 μL of DMSO (Duksan, Korea) was added and the optical density was measured by Biorad microplate reader model 680 (Biorad, USA) at 570 nm.

### Colony formation assay

Hep3B and Huh7 cells transfected with CPSF6 siRNA or negative control siRNA were seeded in six-well cell culture plates at 1 × 10^3^ cells/well to examine the proliferation of CPSF6 depleted cells. Medium of the cells was changed every three days and incubated for two weeks to form colonies under at 37 ℃ 5% CO_2_ incubator. The colonies were stained with Diff Quick solution 2 (Cat No.38721; Sysmex Corporation, Japan), dried overnight, and counted.

### Wound healing assay

Hep3B cells were transfected with siRNA-control and siRNA-CPSF6 for 48 h. A wound-healing assay was then conducted. A scratch was created with a plastic tip in the control or CPSF6 siRNA-transfected Hep3B cells. After 24 h or 48 h incubation, the migratory activity of the cells was measured.

### Cell cycle analysis

Hep3B and Huh7 cells (1 × 10^5^cells/ml) transfected by CPSF6 siRNA or negative control siRNA for 48 h were washed twice with cold PBS and fixed in 75% ethanol at -20°C. The cells were incubated with RNase A (10 mg/mL) for 1 h at 37 °C and stained with propidium iodide (50 μg/mL) for 30 min at 37°C in dark. The DNA content of the stained cells was analyzed by FACSCalibur (Becton Dickinson, Franklin Lakes, NJ, USA) using the Cell Quest Software.

### Quantitative Real-Time polymerase chain reaction* (q*RT-PCR) analysis

To evaluate the expression of CPSF6 (forward primer sequence: 5'-GCTGAATATGGTGGGCATGAT-3', reverse primer sequence: 5'-ATTTGGTGCTGCTCCTTTACC-3'), IL-6 (forward primer sequence: 5'-CCACCGGGAACGAAAGAGAA-3', reverse primer sequence: 5'-GAGAAGGCAACTGGACCGAA-3'), TGF-β (forward primer sequence: 5'-TTGCTGAGGTATCGCCAGGAA-3', reverse primer sequence: 5'-CTACTACGCCAAGGAGGTCA-3'), and GAPDH (forward primer sequence: 5'-AGCCACATCGCTCAGACAC-3', reverse primer sequence: GCCCAATACGACCAAATCC-3'), RT-qPCR analysis was conducted. Total RNAs was isolated from Hep3B cells transfected with siRNA-control or siRNA-CPSF6 plasmids for 48 h using QIAzol (Invitrogen, Carlsbad, CA, USA). The cDNA was synthesized using oligo dT (Bioneer, Daejeon, Korea) and M-MLV reverse transcriptase (Enzynomics, Daejeon, Korea). RT-qPCR was performed using a Light Cycler (Roche, Basel, Switzerland) according to the manufacturer's protocol.

### Tissue microarray and immunohistochemistry

An HCC patient tissue microarray (80 cases) was purchased from Biomax (HLivH160CS01, Derwood, MD, USA), and immunohistochemistry (IHC) staining was performed using Discovery XT (Roche, Mannheim, Germany). HCC and matched normal adjacent tumor tissues in the microarray plate were fixed with 4% paraformaldehyde, dehydrated, embedded in paraffin and sectioned at 4 µm. Sections were deparaffinized, rehydrated, and incubated with 3% H_2_O_2_. After antigen repair and being blocked, the slides were incubated with mouse monoclonal antibodies against CPSF6 (1:200) (sc-376228; Santacruz, Dallas, TX, USA) at 4°C overnight. Subsequently, the slides were incubated with the secondary antibody at room temperature for 30 min and then with a streptavidin peroxidase complex. Staining was performed using 3 a 3-diaminobenzidine (DAB) substrate kit for the peroxidase reaction and counterstained with hematoxylin. Finally, the slides were analyzed under a light microscope.

### Western blotting

Hep3B and Huh7 cells transfected by transfected by CPSF6 siRNA or negative control siRNA were lysed in NP40 buffer containing 50 mM Tris/HCL (pH7.5), 0.5% NonidetP-40, 1 mM EDTA, 120 mM NaCl, 1 mM dithiothreitol, 0.2 mM phenylmethylsufonylfluoride with protease inhibitor cocktails (Roche, Mannheim, Germany) and phosphatase inhibitors (Merck KGaA, Darmstadt, Germany). Lysates were quantified using a DC Protein Assay Kit II (Bio-Rad, Hercules, CA, USA). The protein samples were electrophoresed on 8 to 15% SDS-polyacrylamide gels and transferred to nitrocellulose membranes. Membranes were blocked with Tris Buffered Saline with Tween ® 20 (TBST) diluted 5% skim milk for 1 h at room temperature or TBST diluted 5% bovine serum albumin (BSA) for 4 h at 4 ℃. The cells were incubated with primary antibodies against CPSF6 (Cat. sc-376228.Santacruz, USA), PARP (Cat. No. 9542, Cell Signaling Technology, Danvers, MA, USA), cleaved caspase 3 (Cat No 9664, Cell Signaling Technology, USA), c-Myc (Cat No ab32072, Abcam, USA), FBW7 (Cat No ab109617, Abcam, Waltham, MA, USA), SKP2 (Cat. No. sc-7164, Santacruz Biotechnology, Dallas, TX, USA), Granzyme B (Cat. No. sc-8022, Santacruz Biotechnology, Dallas, TX, USA), HK2 (Cat No 2106, Cell Signaling Technology, USA), PKM2 (Cat. No. 4053, Cell Signaling Technology, USA), LDH (Cat No sc-133123, Santacruz, USA) and β-actin (Cat No A2228, Merck KGaA, USA) diluted in 5% BSA in TBST overnight at 4 ℃, washed thrice for 10 min with TBST, and incubated with HRP-conjugated secondary antibodies (Cell Signaling Technology, USA) for 2 h. The expression was visualized using the ECL Immunoblotting detection reagent (GE Healthcare, Giles, UK).

### Co-Immunoprecipitation

Hep3B cells transfected with CPSF6 siRNA or negative control siRNA were lysed in lysis buffer (50 mM Tris-HCl, pH 7.4, 0.1% SDS, 150 mM NaCl, 1% Triton X-100, 1 mM NaF, 1 mM EDTA, 1 mM Na_3_VO_4_, and 1× protease inhibitor cocktail) and then were immunoprecipitated with antibodies against CPSF6 and c-Myc. Thereafter, protein A/G sepharose beads (Santa Cruz Biotechnology, Santa Cruz, CA) were applied. Precipitated proteins were subjected to immunoblotting using the indicated antibodies.

### Protein-protein interaction prediction

Interactions between CPSF6 and its associated proteins were assessed using the STRING database. All scores ranged from 0 to 1, with 1 indicating the highest possible confidence.

### Immunofluorescence

Hep3B cells transfected with CPSF6 siRNA or negative control siRNA were fixed on poly-L-lysine-coated slides in 4% paraformaldehyde and permeabilized in 0.1% Triton X-100. The permeabilized cells were incubated with 3% BSA in PBS for 1 h, followed by immunostaining with mouse polyclonal CPSF6 (Cat No. sc-376228.Santacruz, USA) and rabbit polyclonal c-Myc (Cat No ab32072, Abcam, USA). Mouse and Rabbit IgG FITC antibodies H & L (Abcam, Cambridge, MA, USA) were used as secondary antibodies. Immuno-stained cells were mounted in a medium containing DAPI (Vectashield, Vector Labs, Burlingame, CA, USA) and visualized under a FLUOVIEW FV10i confocal microscope (Olympus Corporation, Tokyo, Japan).

### Ubiquitination assay

Hek-293T cells transfected with siRNA-Control and siRNA-CPSF6 plasmids (V5-c-Myc and HA-Ubiquitin), followed by addition of 20 μM proteasome inhibitor MG132 for 2 h, and immunoprecipitated with anti-HA antibody and protein G-agarose beads, and immunoblotted with anti-HA antibody.

### Cycloheximide chase assay

Hep3B cells transfected with siRNA-Control and siRNA-CPSF6 plasmids for 48 h were treated with 50 μg/mL cycloheximide (CHX, Merck KGaA, Darmstadt, Germany) for 30, 60, and 120 min or 20 μmol/L MG132 for 4 h before harvest for Western blotting.

### Lactate and Glucose assay

Hep3B cells were transfected with siRNA-control and siRNA-CPSF6 plasmids for 48 h and the cell culture medium was collected. Lactate (K-607) and glucose (K-606) levels (BioVision, Milpitas, CA, USA) were measured using Enzyme-Linked Immunosorbent Assay (ELISA).

### Glucose uptake assay

Glucose uptake was measured using the fluorescent glucose analog 2-NBDG (Cayman Chemicals, Ann Arbor, MI, USA). Cells seeded in glass-bottom dishes were incubated with 100 μM2-NBDG for 2 h. Fluorescent images were taken by confocal microscope (Zeiss LSM 700 microscope) with 488 nm laser as excitation source. Images from approximately ten fields of view were acquired for each sample.

### Tube formation assay

Matrigel (BD Biosciences, Bedford, MA, USA) was thawed at 4 ℃ overnight. The 48 well plates coated with 150 mL Matrigel were incubated at 37 ℃ for 30 min. After gel formation, HUVEC (1 × 10^5^ cells/well) were seeded on a layer of polymerized Matrigel with the culture media from CPSF6 depleted or untreated Hep3B cells. After 6 h incubation, the cells were fixed with 4% formaldehyde and randomly chosen fields were photographed under an Axiovert S 100 light microscope (Carl Zeiss, USA) at 100x magnification. Tube networks were quantified using **National Institutes of Health (**NIH) Scion imaging program.

### Luciferase reporter assay

The VEGF promoter construct was cotransfected into CPSF6 depleted Hep3B cells along with *Renilla* luciferase reporter plasmid. Two days after transfection, the luciferase activity was measured using a Dual-Luciferase Reporter Assay System (Promega, Madison, WI, USA).

### CAM (Chick chorioallantoic membrane) assay

To examine the angiogenic activity of CPSF6, we performed a CAM assay *in vivo*. Briefly, culture media from CPSF6 depleted or untreated Hep3B cells were loaded onto a 1/4 piece of a Thermonix disc (Nunc, Naperville, IL, USA). Discs were applied to the CAM of nine-day-old embryos and incubated for 48 h. The fat emulsion was then injected under the CAM and the number of newly formed blood vessels was photographed and counted. The experiment was repeated for two groups (five eggs per group).

### Synergy validation by combination of CPSF6 depletion and Sorafenib

To determine the synergy between CPSF6 depletion and sorafenib treatment, cytotoxicity assays, cell cycle analysis, and Western blotting were performed on Hep3B cells exposed to si-CPSF6 and/or sorafenib. Combination index of CPSF6 depletion and sorafenib treatment was analyzed using Compusyn software (ComboSyn, Inc., New York, NY, USA) and SynergyFinder 32, 33.

### Establishment of CPSF6 shRNA Hep3B cell lines

To establish Hep3B cell lines stably expressing CPSF6, control shRNA, CPSF6 shRNA lentiviral vectors, and transfection mixtures (TurboFect; Thermo Fisher Scientific, Radnor, PA, USA) were transfected into Hep3B cells. The transfected cells were grown in the medium supplemented with puromycin at 2 µg/mL for approximately 14 days to eliminate the untransfected cells. Subsequently, the macroscopic clones were picked out and continuously cultured in the medium supplemented with puromycin (0.5 -1.5 ng/mL). CPSF6 protein expression was evaluated by Western blotting in Hep3B cells transfected with control shRNA and CPSF6 shRNA.

### Liver tumor orthotopic & xenograft models and immunohistochemistry

All animal experiments were approved by the Institutional Animal Care and Use Committee of the Kyung Hee University (KHUASP-21-205). For the xenograft model, 1x10^6^ of LV-shControl or LV-shCPSF6 Hep3B cells were suspended in a serum-free medium and mixed with Matrigel (Corning, Bedford, MA, USA) in a 1:1 ratio. The cell mixture was injected subcutaneously into the right flank of athymic BALB/c mice. All mice were euthanized 42 d after implantation, and the isolated tumors were photographed and weighed. Flow cytometry analysis was conducted to determine the percentage of CD8/CD4 in splenocytes isolated from the mice.

For the orthotopic tumor model, 2 ×10^6^ of LV-shControl or LV-shCPSF6 Hep3B cells were suspended in serum-free medium and mixed with Matrigel matrix (Corning, Bedford, MA, USA) at a 1:1 ratio. Using a 31G insulin syringe, a small scratch was made in the left-lateral lobe (s) of the livers of BALB/c nude mice under anesthesia with 2% isoflurane, and the cell mixture was slowly injected. The incision site was closed with sutures as previously described 34. All the mice were euthanized 42 d after implantation. Immunohistochemistry was performed on tumor sections using antibodies against CPSF6 (Cat No. sc-376228. Santa Cruz, USA), anti-c-Myc (cat no. sc-40. Santacruz, USA), PCNA (Cat No. sc-56; Santa Cruz, USA), Caspase3 (Cat. No. 9662; Cell Signaling Technology, USA), CD4 (Cat No. sc-13573. Santa Cruz, USA), and CD8 (Cat No. sc-1181. Santa Cruz, USA), HK2 (Cat. No. 2106, Cell Signaling Technology, USA), PKM2 (Cat. No. 4053, Cell Signaling Technology), anti-LDH (Cat No. sc-133123. Santa Cruz, USA), VEGF (Cat No. sc-152. Santa Cruz, USA), and PD-L1 (Cat No.17952-1-AP, Proteintech, USA).

### Statistical analysis

SigmaPlot version 12 software (Systat Software Inc., San Jose, CA, USA) was used for statistical analysis. All data were expressed as means ± standard deviation (SD). Student's t-test was used to compare two groups. One-way analysis of variance (ANOVA) followed by Tukey's post-hoc test was performed for multigroup comparisons using GraphPad Prism software (Version 5.0, San Diego, CA, USA). p < 0.05 between the control and treated groups were considered statistically significant.

## Results

### Overexpression of CPSF6 in HCC patient tissues and cytotoxic and apoptotic effects of CPSF6 depletion in HCCs

**Figure 1:**
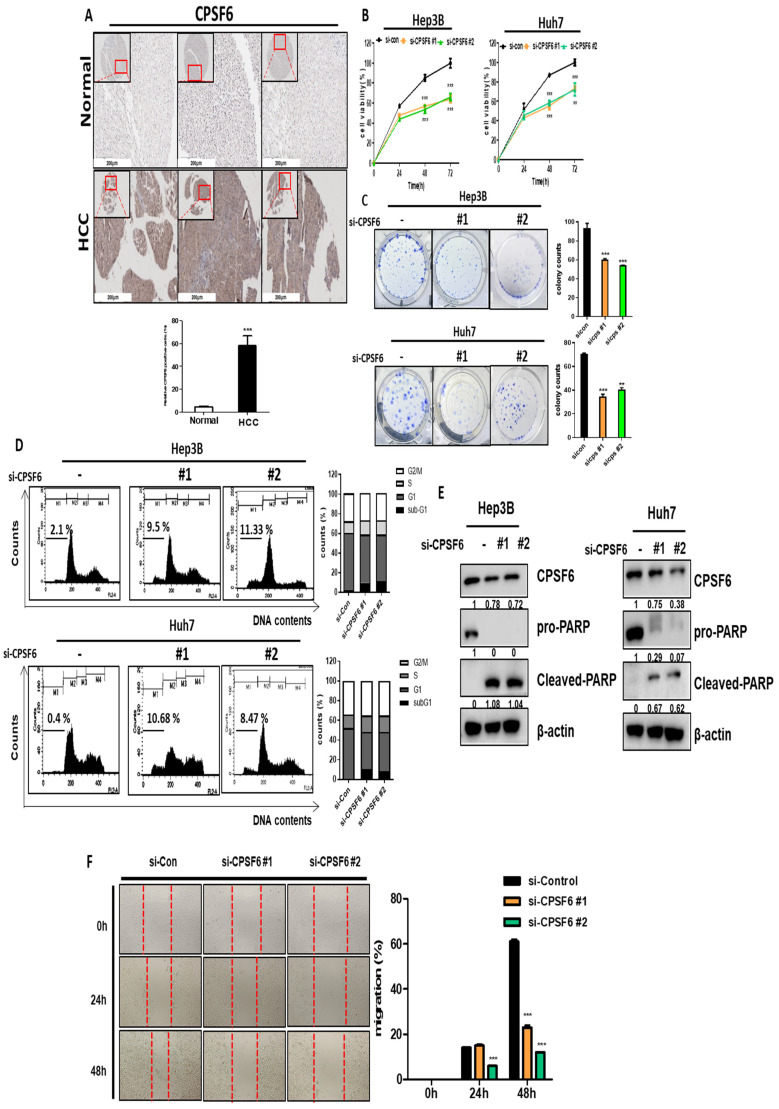
** Overexpression of CPSF6 in HCC tissues and cytotoxic and apoptotic effect of CPSF6 depletion in HCCs.** (A) Overexpression of CPSF6 in human HCC tissues. CPSF6 was overexpressed in human hepatocellular carcinoma tissues compared to adjacent normal tissues by immunohistochemistry. Data represent means ± SD. ***p < 0.001 vs untreated control. (B) Effect of CPSF6 depletion on the viability of Hep3B and Huh7 cells transfected with control vector or CPSF6 siRNA plasmid by MTT assay. ***p < 0.001 vs untreated control. (C) Effect of CPSF6 depletion on the number of colonies in Hep3B and Huh7 cells transfected with control vector or CPSF6 siRNA plasmid by colony formation assay. ** p < 0.01, ***p < 0.001 vs untreated control. (D) Effect of CPSF6 depletion on sub G1 population in Hep3B and Huh7 cells transfected with control vector or CPSF6 siRNA plasmid. (E) Effect of CPSF6 depletion on PARP in Hep3B and Huh7 cells transfected with control vector or CPSF6 siRNA plasmid. (F) Effect of CPSF6 depletion on the migratory activity of Hep3B cells transfected with control vector or CPSF6 siRNA plasmid by Wound healing assay. ***p < 0.001 vs untreated control. Experiments were performed in triplicate and repeated three times.

The human tissue array showed overexpression of CPSF6 in HCC patient tissues compared to adjacent normal liver tissue (Figure 1A). In addition, TCGA analysis revealed highly mRNA expression of CPSF6 in HCC patients, with a poor overall survival rate compared to normal controls (Figure S1A-B). Consistently, CPSF6 was overexpressed in stages Ⅰ-Ⅵ of HCC (Figure S1C) and was highly expressed at the mRNA level in various cancer cell lines, including breast, liver, colon, and prostate cancers (Figure S1D). To assess the cytotoxic and anti-proliferative effects of CPSF6 depletion in HCCs, MTT and colony formation assays were conducted in Hep3B and Huh7 cells. As shown in Figure 1B, CPSF6 depletion induced significant cytotoxicity in Hep3B and Huh7 cells. Similarly, the colony formation assay revealed that the number of colonies was significantly reduced in CPSF6 depleted HCCs compared with that in untreated controls (Figure 1C). To confirm the cytotoxicity by CPSF6 depletion is due to apoptosis, cell cycle analysis and western blotting were conducted for PARP, CPSF6, and β-actin in Hep3B and Huh7 cells. CPSF6 depletion increased the sub-G1 cell population and cleaved PARP in Hep3B and Huh7 cells (Figure 1D-E). Furthermore, CPSF6 depletion inhibited the migratory activity of Hep3B cells in a wound healing assay (Figure 1F). According to the gene profile and related signaling pathways determined by microarray analysis, there were differentially expressed gene profiles and signaling pathways in CPSF6 silenced cells (Figure S2A-B). Gene ontology analysis classified the affected genes and signaling pathways as apoptosis, cell cycle, cell migration, cell proliferation, cell migration, cell cycle, and angiogenesis (Figure S2B). Furthermore, metabolism-related genes (22%), cell cycle-related genes (14%), cell death- related genes (12%), and apoptosis-related genes (10%) were critically involved in CPSF6-depleted Hep3B cells (Figure S2C). Metabolism-related genes were differentially expressed upon upregulation (red) or downregulation (blue) (Figure S2D). Hence, further studies have been conducted on cancer metabolism, angiogenesis, and immune escape from TME.

### CPSF6 modulates c-Myc in HCCs through their binding and co-localization

**Figure 2:**
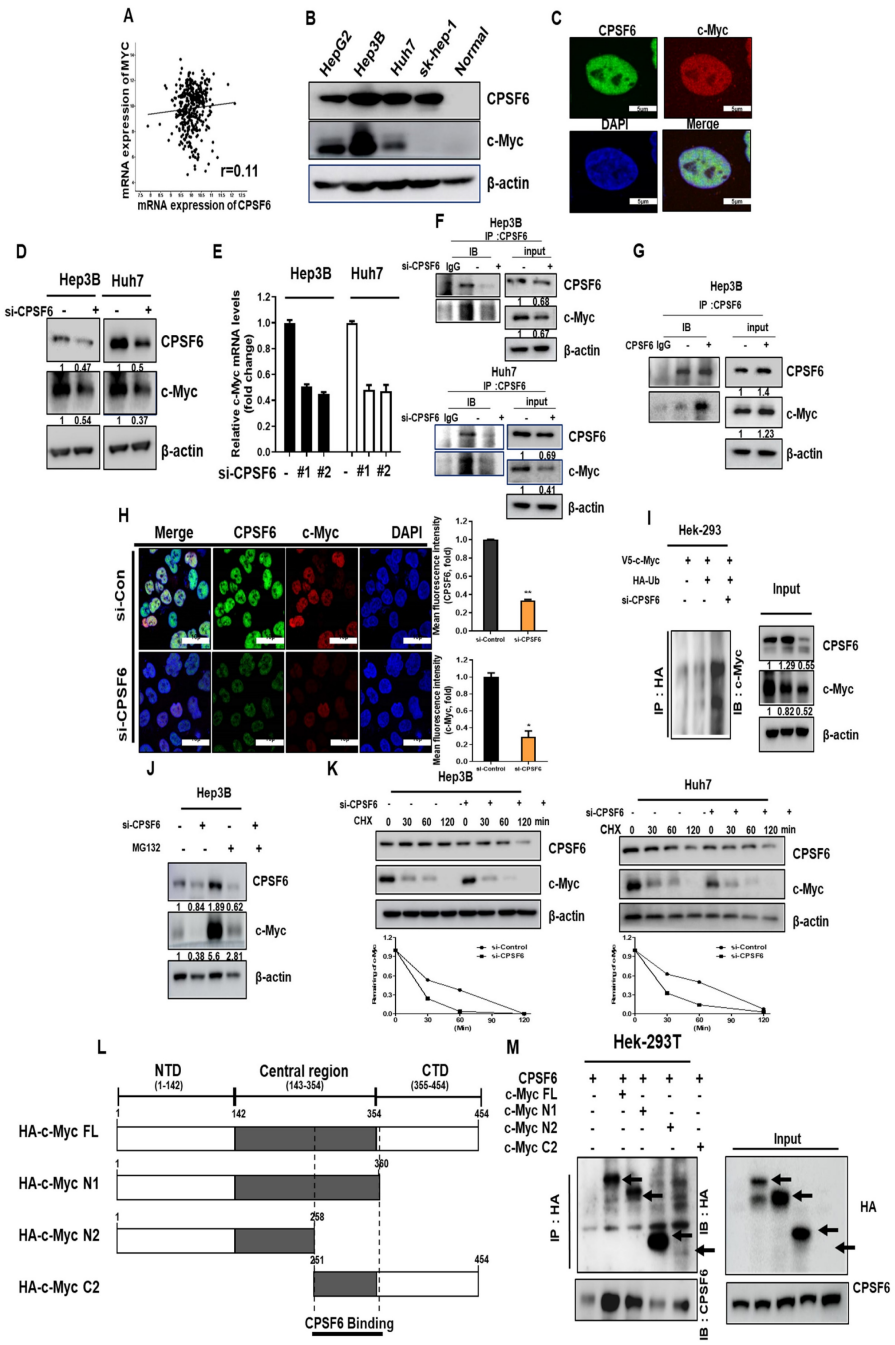
** CPSF6 regulated c-Myc in HCCs through their binding and colocalization**. (A) Spearman's correlation coefficient (*r* = 0.11) between CPSF6 and c-Myc, using TCGA database. (B) Endogenous protein expression levels of CPSF6 and c-Myc in HCCs and normal human liver lysates (NLH-1, G-Biosciences, MO, USA) by Western blotting. (C) Colocalization of CPSF6 and c-Myc in Hep3B cells by immunofluorescence (c-Myc, red; CPSF6, green; DAPI, blue). (D) Effect of CPSF6 depletion on c-Myc expression in Hep3B and Huh7 cells as determined by Western blotting. (E) Effect of CPSF6 depletion on c-Myc mRNA expression in Hep3B and Huh7 cells, as determined by qRT-PCR. (F) Binding between CPSF6 and c-Myc in CPSF6 depleted Hep3B cells as determined by IP. Hep3B and Huh7 cells transfected with control or CPSF6 siRNAs were subjected to IP and Western blotting. (G) Binding between CPSF6 and c-Myc in Hep3B cells as determined by IP. Hep3B and Huh7 cells transfected with the control vector and the CPSF6 plasmid were subjected to IP and Western blotting. (H) Effect of CPSF6 depletion on c-Myc in Hep 3B cells by IF. Hep3B cells transfected with the control vector or CPSF6 siRNA were subjected to immunofluorescence staining for c-Myc, CPSF6, and DAPI (c-Myc in red; CPSF6 in green; DAPI in blue). * p < 0.05, **p < 0.01 vs untreated control. (I) The Effect of CPSF6 depletion on c-Myc degradation in Hep3B cells. Hek-293T cells transfected with control vector, V5-tagged c-Myc, HA-tagged ubiquitin, and CPSF6 siRNA plasmids were subjected to Immunoprecipitation with anti-HA antibody detected by c-Myc antibody, while β-actin was immunoblotted as a loading control. (J) The effect of the proteasome inhibitor MG132 on c-Myc expression in CPSF6 depleted Hep3B cells. Hep3B cells transfected with CPSF6 siRNA plasmid were exposed to MG132 for 5 h for Western blotting. (K) The Effect of CPSF6 depletion on c-Myc stability in CPSF6 depleted Hep3B and Huh7 cells in the presence or absence of the protein synthesis inhibitor cycloheximide. (L) The full-length and three-fragment domains of c-Myc. (M) CPSF6 bound to c-Myc in Hek-293T cells transfected with CPSF6 and c-Myc domain plasmids.

To assess the correlation between CPSF6 and c-Myc, cBioPortal database, Western blotting, and Immunoprecipitation and Immunofluorescence analyses were performed. Despite the weak correlation between CPSF6 and c-Myc with Spearman's correlation coefficient (r = 0.11) using the cBioPortal database (Figure 2A), the endogenous expression of CPSF6 was similar to that of c-Myc in HCCs cells, such as HepG2, Hep3B, Huh7, and Sk-Hep1 cells (Figure 2B). Colocalization of CPSF6 and c-Myc was observed in the nucleus rather than in the cytoplasm of Hep3B cells using immunofluorescence (Figure 2C). Consistently, CPSF6 depletion decreased c-Myc expression at protein (Figure 2D) and mRNA levels (Figure 2E) in Hep3B and Huh7 cells. Immunoprecipitation validated the binding between CPSF6 and c-Myc in Hep3B cells, regardless of depletion or overexpression of CPSF6 (Figure 2F-G). CPSF6 (green) depletion decreased c-Myc (red) in Hep3B cells (Figure 2H). Furthermore, CPSF6 depletion enhanced HA-Ub-induced c-Myc degradation in Hek-293T cells. (Figure 2I). In addition, the proteasomal inhibitor MG132 reversed the reduced expression of c-Myc induced by CPSF6 depletion in Hep3B cells (Figure 2J). The half-life of c-Myc protein was remarkably reduced by CPSF6 depletion in the presence of protein synthesis inhibitor cycloheximide in Huh7 and Hep3B cells (Figure 2K), Notably, among the three domains of c-Myc including HA-N1 domain (101-360), HA-N2 domain (1-258) and HA-C2 domain (251-454) (Figure 2L), Immunoprecipitation revealed that CPSF6 binds directly to c-Myc at the site of 258-360 in Hek-293T cells cotransfected with CPSF6 and three domain plasmids of c-Myc (Figure 2M).

### CPSF6 depletion induces c-Myc degradation mediated by FBW7 in HCCs

**Figure 3:**
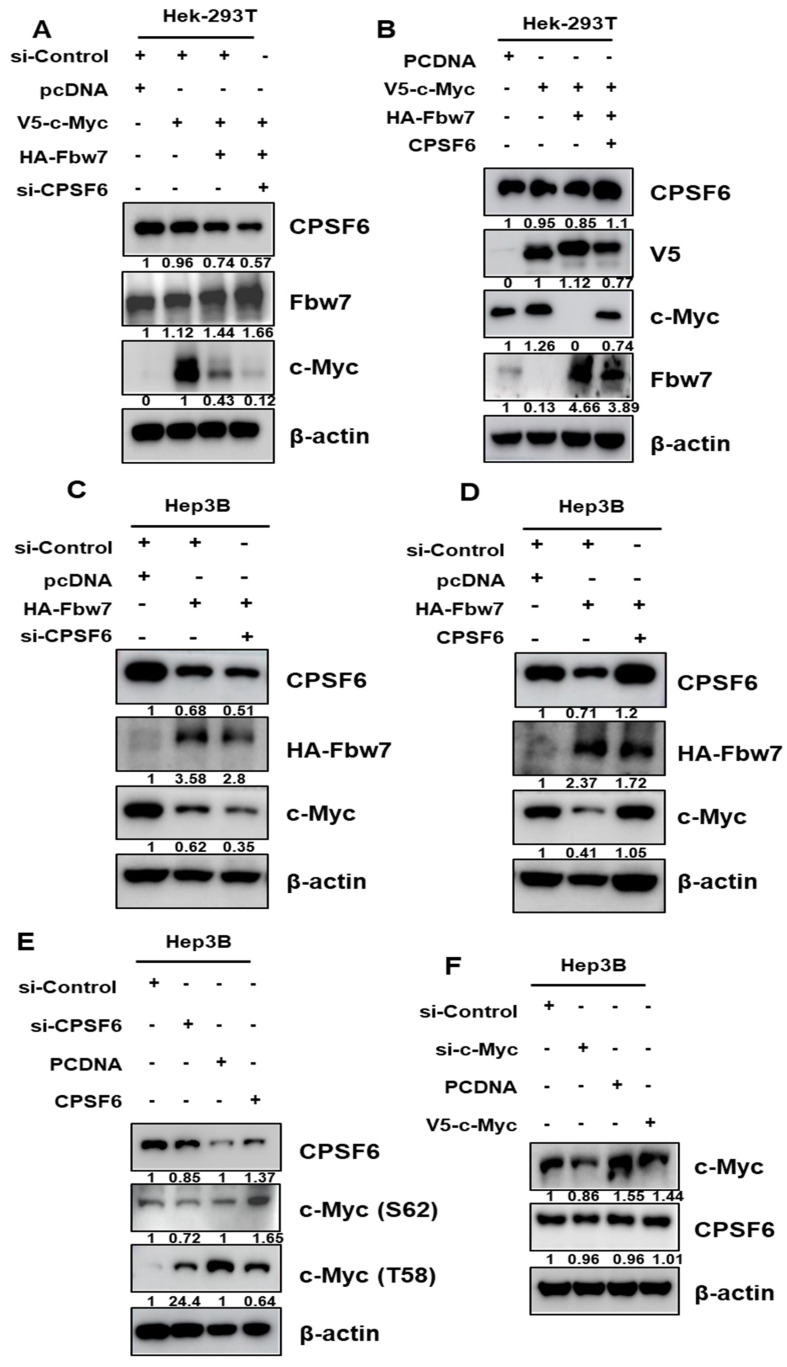
** CPSF6 depletion enhanced c-Myc degradation in HCCs.** (A, B) Effect of FBW7 overexpression on c-Myc expression in Hep3B and Hek-293T cells. Control vector, V5-tagged c-Myc, HA-tagged FBW7 and CPSF6 siRNA plasmids were cotransfected into Hek-293T cells for Western blotting. (C) Effect of FBW7 overexpression on c-Myc expression in CPSF6 depleted Hep3B cells. (D) Effect of FBW7 overexpression on c-Myc expression in CPSF6 overexpressed Hep3B cells. (E) Effect of CPSF6 depletion or overexpression on c-Myc (S62) and c-Myc (T58) expression in Hep3B cells. (F) Effect of c-Myc depletion or overexpression on CPSF6 expression in Hep3B cells. Experiments were performed in triplicate and repeated three times.

Since E3 ubiquitin ligase participates in c-Myc degradation 35, c-Myc ubiquitin ligases such as FBW7 and SKP2 were transfected into Hep3B cells. Here, FBW7 overexpression enhanced c-Myc degradation in CPSF6 depleted Hep3B cells (Figure 3A), whereas FBW7 depletion restored the expression of c-Myc, whereas SKP2 depletion did not (Figure S3). However, CPSF6 overexpression reduced the ability of FBW7 to inhibit c-Myc expression in Hek-293T cells (Figure 3B). Furthermore, depletion or overexpression of CPSF6 regulated FBW7 induced degradation of endogenous c-Myc in Hep3B cells (Figure 3C-D). Since S62 phosphorylation of c-Myc enhances Myc-driven oncogenic cascades and T58 phosphorylation induces Myc degradation through the ubiquitin-proteasome system, depletion or overexpression of CPSF6 regulates the phosphorylation of c-Myc (S62) and c-Myc (T58) (Figure 3E) 36. In contrast, depletion or overexpression of c-Myc did not affect the expression of CPSF6 (Figure 3F), implying that CPSF6 acts upstream of c-Myc.

### CPSF6 depletion suppresses glycolysis, and its related genes in HCCs

**Figure 4:**
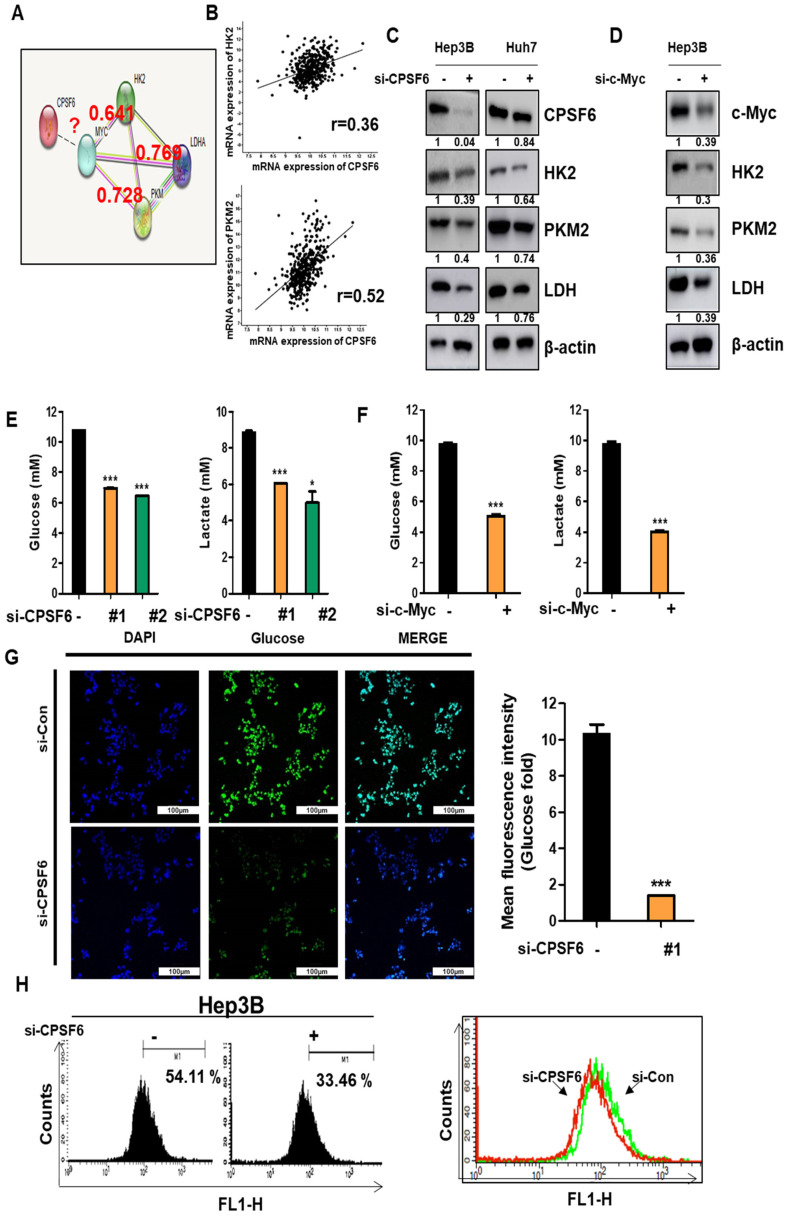
** CPSF6 depletion suppressed cancer metabolism related genes, glucose and lactate in HCCs.** (A) Protein-protein interaction between CPSF6, c-Myc, HK2, and PKM by STRING database. (B) A strong correlation between CPSF6 and HK2 or PKM2 by Spearman's correlation coefficients (*r* = 0.36, and *r* = 0.52, respectively) by cBioPortal database. (C) Effect of CPSF6 depletion on HK2, PKM2 and LDH in Hep3B and Huh7 cells by Western blotting. (D) Effect of c-Myc depletion on HK2, PKM2 and LDH in Hep3B cells by Western blotting. (E) Effect of CPSF6 depletion on glucose and lactate in the culture supernatants from Hep3B cells by ELISA. *p < 0.05, ***p < 0.001 vs untreated control. (F) Effect of c-Myc depletion on glucose and lactate in the culture supernatants from Hep3B cells by ELISA. ***p < 0.001 vs untreated control. (G) Effect of CPSF6 depletion on glucose uptake in Hep3B cells by Immunofluorescence. (H) Effect of CPSF6 depletion on glucose uptake in Hep3B cells by flow cytometry analysis. Data represent means ± SD. Experiments were performed in triplicate and repeated three times.

Based on the mRNA-Sequencing data from CPSF6 depleted Hep3B cells (Figure S2C-D), the effect of CPSF6 depletion on glucose metabolism was explored in HCCs by Western blotting, ELISA, Flow cytometry, and immunofluorescence. Regarding protein-protein interactions, the binding scores between c-Myc and HK2, PKM2, or LDH were 0.642, 0.728, and 0.769, respectively, using the STRING database (Figure 4A). Moreover, Spearman correlation coefficients between CPSF6 and HK2 or PKM2 were 0.36 and 0.52, respectively (Figure 4B). Western blot revealed that CPSF6 depletion suppressed the protein expression of HK2, PKM2, and LDH in Hep3B and Huh7 cells (Figure 4C). Similarly, the depletion of c-Myc downstream of CPSF6 suppressed HK2, PKM2, and LDH protein expression in Hep3B cells (Figure 4D).

Furthermore, depletion of CPSF6 or c-Myc decreased the levels of glucose and lactate in the culture supernatants of Hep3B cells, as determined by ELISA (Figure 4E-F). Consistent with this, immunofluorescence and flow cytometry analyses showed that CPSF6 depletion reduced glucose uptake in Hep3B cells (Figure 4G-H). However, the depletion of HK2, LDH, PD-L1, and VEGF did not affect the expression of CPSF6 in Hep3B cells (Figure S5), indicating that CPSF6 acts upstream of HK2, LDH, PD-L1, and VEGF.

### CPSF6 depletion suppresses immune escape and its related genes in HCCs

**Figure 5:**
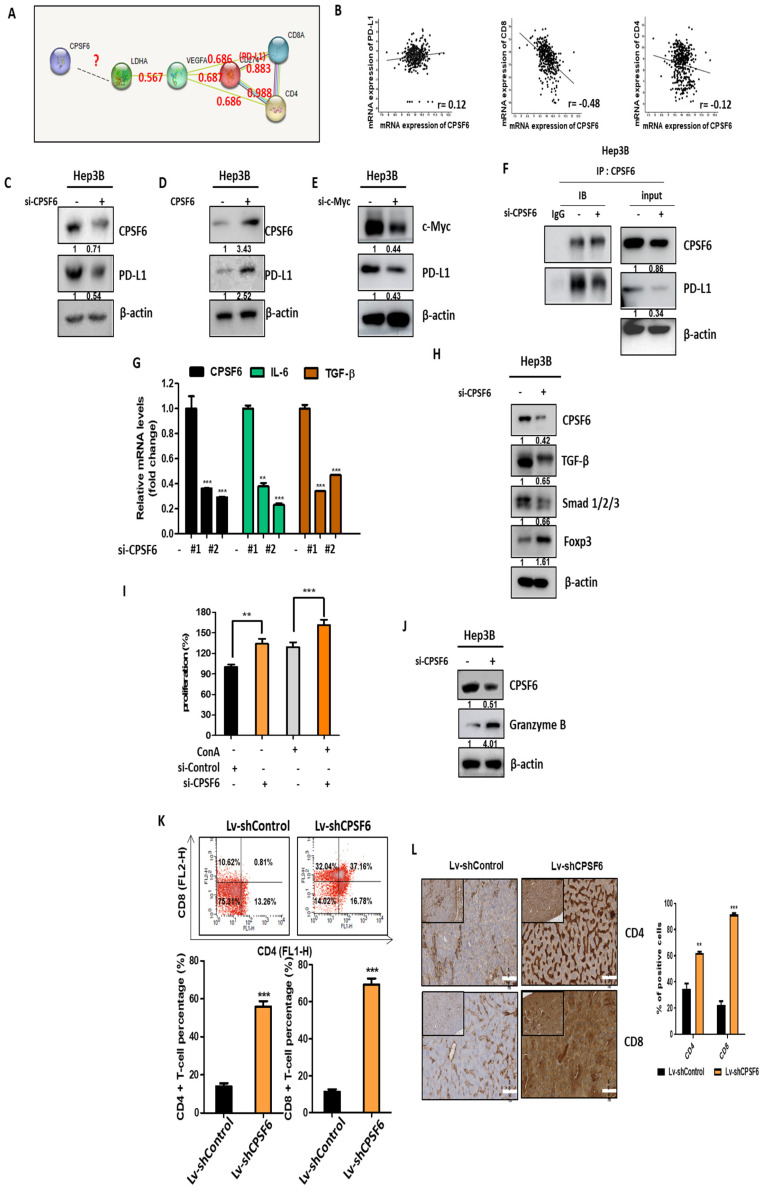
** CPSF6 depletion suppressed immune escape and its related genes in HCCs.** (A) Protein-protein interaction between PD-L1 and CD4 or CD8 by STRING database. (B) Close correlation between CPSF6 and PD-L1(r = 0.12), and inverse correlation between CPSF6 and CD8 (r = -0.48), or CD4(r = -0.12) by Spearman's correlation coefficient. (C) Effect of CPSF6 depletion on the expression of PD-L1 in Hep3B cells. (D) Effect of CPSF6 overexpression on the expression of PD-L1 in Hep3B cells. (E) Effect of c-Myc depletion on the expression of PD-L1 in Hep3B cells. (F) The binding between CPSF6 and PD-L1 in CPSF6 depleted Hep3B cells by IP. (G)Effect of CPSF6 depletion on mRNA levels of IL-6 and TGF-β in Hep3B cells by qRT-PCR. **p < 0.01, ***p < 0.001 vs untreated control. (H) Effect of CPSF6 depletion on protein expression of TGF-β, SMAD1/2/3 and FOXP3 in Hep3B cells. (I) Effect of CPSF6 depletion on the proliferation of murine splenocytes in the presence or absence of T cell activator Concanavalin A (Con A). **p < 0.01, ***p < 0.001 vs untreated control. (J) Effect of CPSF6 depletion on the protein expression of Granzyme B in Hep3B cells. (K) Effect of CPSF6 depletion on the percentage of CD4 and CD8 cells in the splenocytes of BALB/C mice bearing CPSF6 depleted Hep3B cells. The splenocytes of BALB/C mice were isolated six weeks after implantation of CPSF6 depleted Hep3B cells. The percentages of CD4 and CD8 cells were measured in the isolated splenocytes of BALB/c nude mice bearing Hep3B cells transfected with control vector or CPSF6 shRNA plasmid by using flow cytometry. ***p < 0.001 vs untreated control. (L) Effect of CPSF6 depletion on the expression of CD4 and CD8 cells in the tumor section of CPSF6 depleted Hep3B cells inoculated in BALB/C mice by IHC.

Given that the metabolic product lactate is involved in the immune escape of tumor cells in the tumor microenvironment 37, we can expect that CPSF6 may regulate genes related to immune escape. Indeed, a close correlation between PD-L1 and VEGF, CD4, and CD8 was predicted using the STRING database (Figure 5A). Likewise, Spearman's correlation coefficient data reveal that CPSF6 has positive correlation with PD-L1 (r = 0.12), while it has inverse correlation with CD8 (r = -0.48) or CD4 (r = -0.12) (Figure 5B). In addition, the depletion or overexpression of CPSF6 attenuated or increased the expression of PD-L1 in Hep3B cells, respectively (Figure 5C-D). Furthermore, the depletion of c-Myc downstream of CPSF6 downstream reduced the expression of PD-L1 in Hep3B cells (Figure 5E). Immunoprecipitation verified the direct binding between CPSF6 and PD-L1 (Figure 5F). Additionally, CPSF6 deletion abrogated mRNA expression of IL-6 and TGF-β as proinflammatory and immune evasion related proteins (Figure 5G) and also suppressed protein expression of TGF-β and SMAD1/2/3 along with activation of a tumor suppressor FOXP3 in Hep3B cells (Figure 5H). Notably, the culture supernatant from CPSF6 depleted Hep3B cells significantly enhanced the proliferation of splenocytes from BALB/C mice in absence or presence of Con A (2 μg/mL) compared to untreated control (Figure 5I). In addition, CPSF6 depletion increased the expression of Granzyme B in Hep3B cells (Figure 5J). Granzyme B is known to mediate cellular apoptosis as a serine proteinase expressed in activated memory CD8 +, memory CD4 + T cells, and NK cells 38. Consistently, the percentage of CD4/CD8 was significantly increased in the splenocytes of BALB/c mice bearing CPSF6 depleted Hep3B cells compared to that in the untreated control (Figure 5K). Furthermore, immunohistochemistry showed that the expression of CD4/CD8 was significantly increased in the tumor tissues of CPSF6 depleted Hep3B cells inoculated in BALB/c mice compared to that in the untreated control (Figure 5L).

### CPSF6 depletion abrogates angiogenesis in HCCs

**Figure 6:**
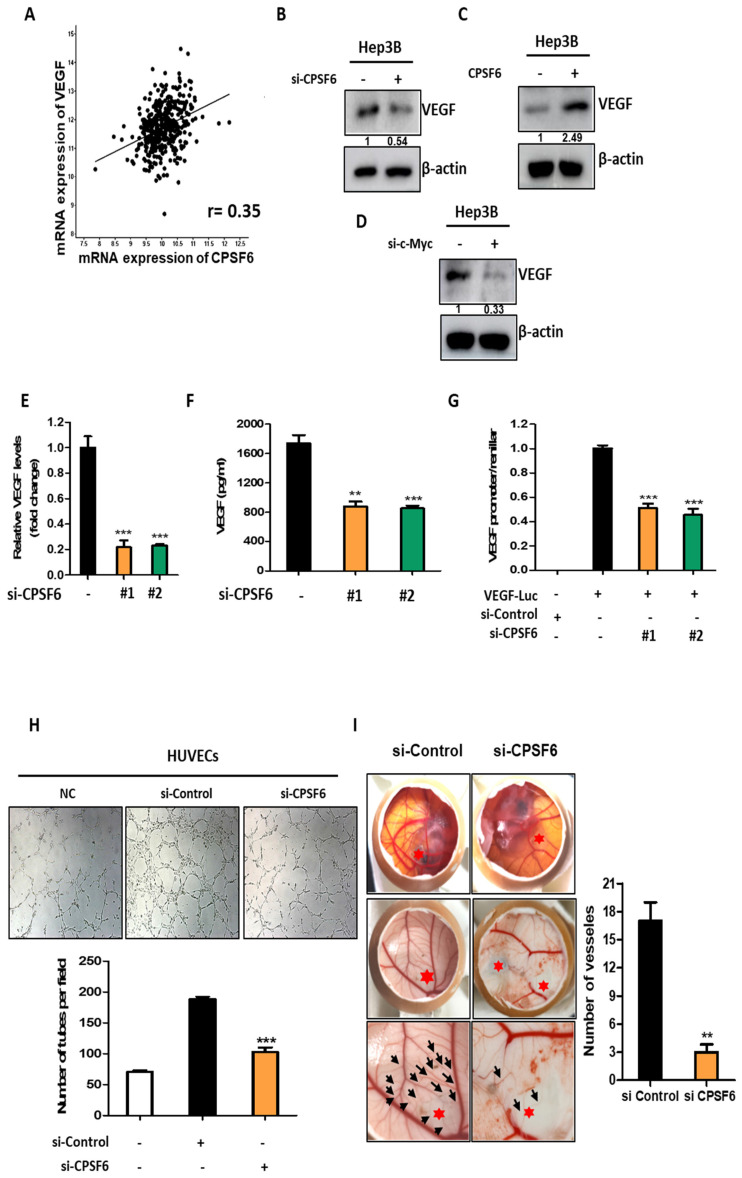
** CPSF6 depletion abrogated angiogenesis in HCCs.** (A) Close correlation between CPSF6 and VEGF by Spearman's correlation coefficient (r = 0.35). (B) Effect of CPSF6 depletion on VEGF in Hep3B cells by Western blotting. (C) Effect of CPSF6 overexpression on VEGF in Hep3B cells by Western blotting. (D) Effect of c-Myc depletion on VEGF in Hep3B cells by Western blotting. (E) Effect of CPSF6 depletion on mRNA level of VEGF in Hep3B cells transfected with CPSF6 siRNA plasmid by qRT-PCR. ***p < 0.001 vs untreated control. (F) Effect of CPSF6 depletion on VEGF production in CPSF6 depleted Hep3B cells by ELISA. **p < 0.01, ***p < 0.001 vs untreated control. (G) Effect of CPSF6 depletion on VEGF activity in Hep3B cells transfected with VEGF-Luc reporter by luciferase assay. ***p < 0.001 vs untreated control. (H) Effect of CPSF6 depletion on the number of tube formed networks in HUVECs compared to untreated control. Tube formation assay was conducted with the culture supernatants from Hep3B cells transfected by CPSF6 siRNA or negative control siRNA. ***p < 0.001 vs untreated control. (I) Effect of CPSF6 depletion on the number of neovascularization networks in CAMs. CAM assay was conducted with the culture supernatants from Hep3B cells transfected by CPSF6 siRNA. **p < 0.01 vs untreated control. Experiments were performed in triplicate and repeated three times.

The cBioPortal database showed a close correlation between CPSF6 and VEGF using Spearman's correlation coefficient (r = 0.35) (Figure 6A). Here, the depletion or overexpression of CPSF6 attenuated or increased VEGF expression in Hep3B cells, respectively (Figure 6B-C). Furthermore, c-Myc depletion suppressed the protein expression in Hep3B cells (Figure 6D). In addition, CPSF6 depletion significantly decreased the mRNA level of VEGF in Hep3B cells compared to the untreated control (Figure 6E) and significantly reduced VEGF production in the culture supernatants from CPSF6 depleted Hep3B cells compared to the untreated control (Figure 6F). Consistently, CPSF6 depletion significantly reduced VEGF promoter luciferase activity compared to that in the untreated control in Hep3B cells transfected with the VEGF-Luc reporter (Figure 6G). To validate the angiogenic activity of CPSF6, tube formation and CAM assays were conducted using culture supernatants from Hep3B cells transfected with CPSF6 siRNA or negative control siRNA. The tube formation assay showed that CPSF6 depletion significantly reduced the number of tube-formed networks in HUVECs compared to that in the untreated control (Figure 6H). Similarly, the CAM assay revealed that CPSF6 depletion significantly reduced the number of neovascularization networks in CAMs compared to the untreated control (Figure 6I).

### CPSF6 depletion enhanced antitumor effect of Sorafenib in Hep3B cells

**Figure 7:**
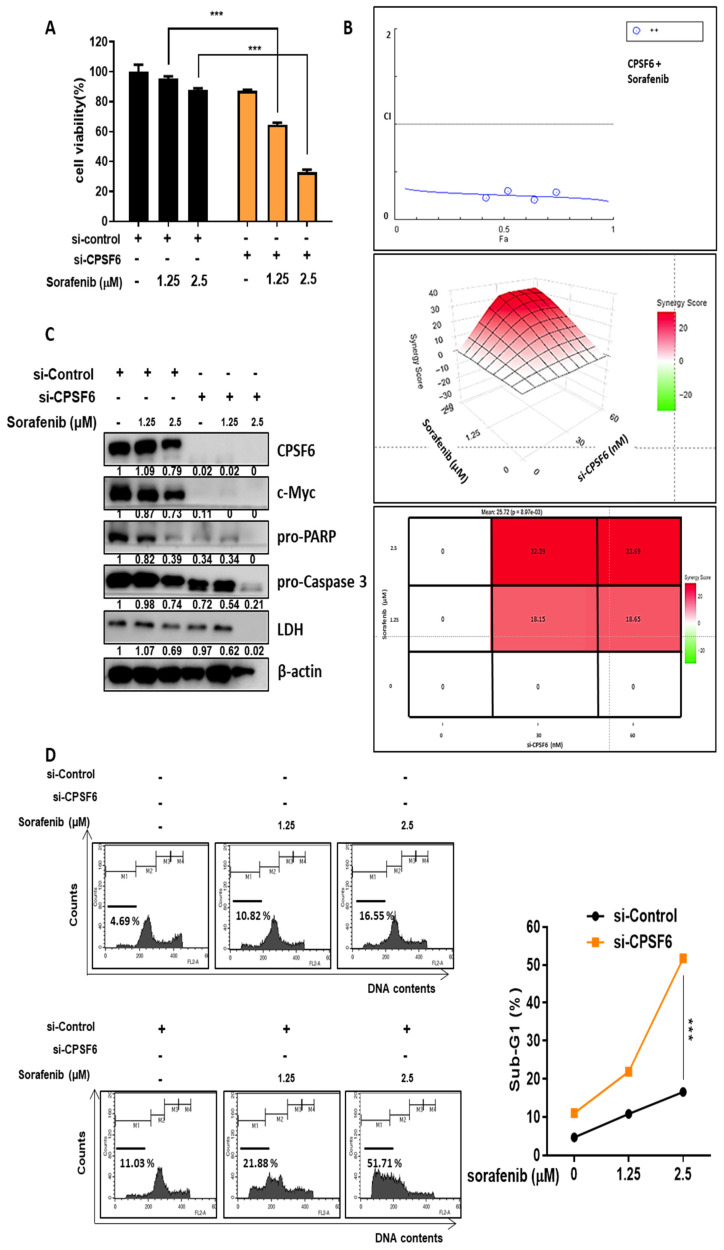
** Synergistic potential of CPSF6 depletion with Sorafenib in Hep3B cells.** (A) Effect of CPSF6 depletion on the cytotoxicity of Sorafenib in Hep3B cells. ***p < 0.001 vs untreated control. (B) Synergy analysis between CPSF6 depletion and Sorafenib by CompuSyn and SynergyFinder softwares. Cytotoxicity data by CPSF6 depletion and/or Sorafenib were analyzed by using CompuSyn and SynergyFinder software. Synergy is determined when the value is below 1 of CI, and is considered synergistic with the score over 10 (red fraction). (C) Effect of CPSF6 depletion and Sorafenib on the expression of c-Myc, pro-PARP, pro-caspase 3 and LDH in Hep3B cells. (D) Effect of CPSF6 depletion and Sorafenib on sub G1 population in Hep3B cells. ***p < 0.001 vs untreated control.

To check the synergistic potential of CPSF6 depletion with Sorafenib, MTT assay, western blotting, and cell cycle analysis were conducted in Hep3B cells. Herein, CPSF6 depletion significantly enhanced the cytotoxicity of Sorafenib in Hep3B cells (Figure 7A), which was confirmed by CompuSyn analysis that the combination index (CI) was below 1, and by SynergyFinder showing a red color above 10 between CPSF6 depletion and sorafenib (Figure 7B). Consistently, CPSF6 depletion enhanced the inhibitory effect of sorafenib on c-Myc, pro-PARP and pro-caspase 3 compared to sorafenib alone in Hep3B cells (Figure 7C). Similarly, CPSF6 depletion significantly increased the G1 population to 21.88% and 51.71% with sorafenib compared to sorafenib alone (10.82% and 16.55%) at 1.25 and 2.5 μΜ, respectively) in Hep3B cells (Figure 7D).

### CPSF6 depletion suppressed the growth of Hep3B cells in orthotopic and xenograft tumor models

**Figure 8:**
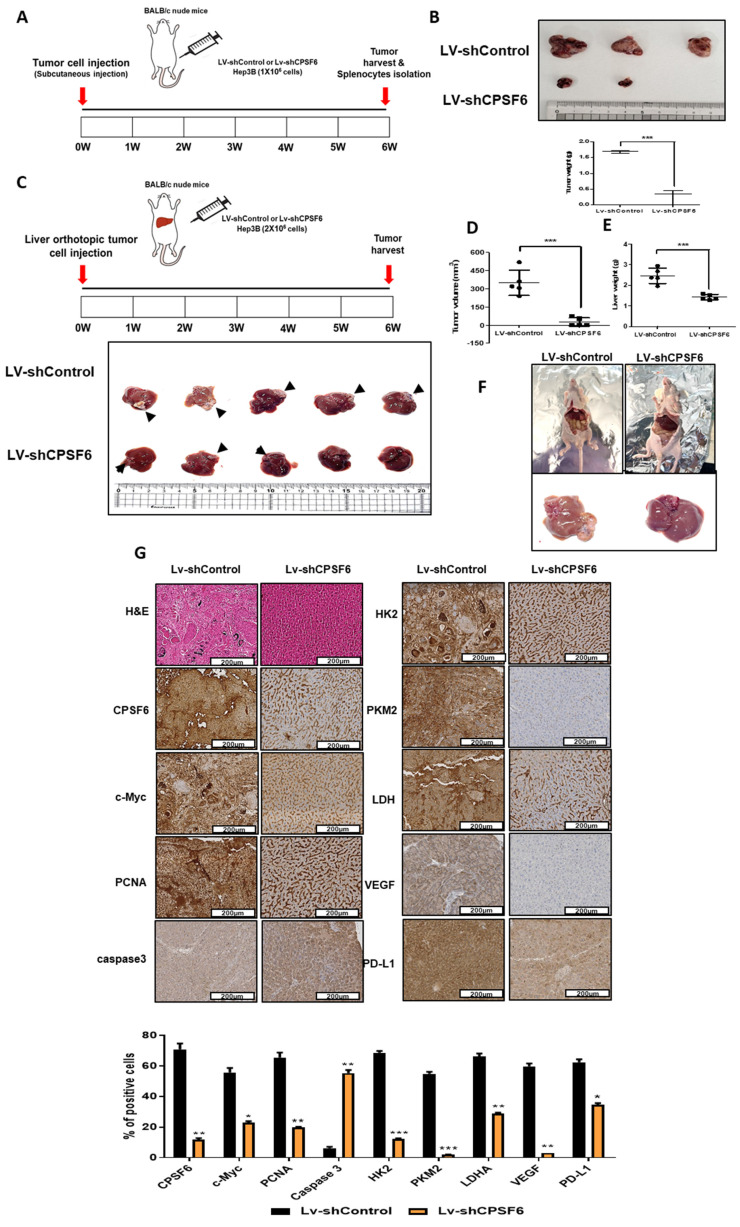
** CPSF6 depletion suppressed the growth of Hep3B cells in orthotopic and xenograft tumor models.** (A) Animal study plan in a xenograft tumor model. Hep3B cells transfected with LV-shControl vector and shCPSF6 plasmid were injected into the right flank of BALB/c athymic nude mice. Six weeks after implantation, tumors and spleens were isolated from the mice for next experiments. (B) Effect of CPSF6 depletion on the volumes of Hep3B cells in a xenograft tumor model. (C)Animal study plan in an orthotopic tumor model. LV-shControl or LV-shCPSF6 Hep3B cells were injected into the left-lateral lobes of the liver of BALB/c nude mice. On Day 42 after implantation, livers including tumors from the sacrificed mice were isolated, photographed and weighed for next experiments. (D) Effect of CPSF6 depletion on the volumes of Hep3B cells in an orthotopic tumor model. ***p < 0.001 vs untreated control. (E) Effect of CPSF6 depletion on liver weights in an orthotopic tumor model. ***p < 0.001 vs untreated control. (F) Morphology of liver and tumors in LV-shControl and LV-shCPSF6 Hep3B groups. (G) Effect of CPSF6 depletion on histopathological changes by H&E staining and the expression of CPSF6, c-Myc, PCNA, caspase 3, HK2, PKM2, LDH, VEGF and PD-L1 by IHC in tumor sections compared to LV-shControl group. *p < 0.05, **p < 0.01, ***p < 0.001 vs untreated control.

To confirm the above-mentioned *in vitro* results, animal studies were conducted in orthotopic and xenograft tumor models of Balb/c male athymic nude mice bearing CPSF6 LV-shRNA- or LV-shControl-transfected Hep3B cells for six weeks (Figure 8A and 8C). Before the animal study, CPSF6 LV-shRNA activity was confirmed in Hep3B cells by immunoblotting for c-Myc, HK2, PKM2, and LDH, and MTT assays compared to the untreated control (Figure S4A-B). In a xenograft tumor model, the growth of Hep3B cells implanted into the right flank of BALB/c nude mice was significantly suppressed in the CPSF6 LV-shRNA-treated Hep3B group compared to that in the LV-shControl group (Figure 8B). Similarly, in an orthotopic tumor model, by direct injection of Hep3B cells into the livers of Balb/c nude mice, CPSF6 depletion significantly reduced the volumes of Hep3B cells and liver weights compared to the sham control group (Figure 8C-F). Furthermore, Immunohistochemistry revealed that CPSF6 depletion decreased the expression of CPSF6, c-Myc, PCNA, HK2, PKM2, LDH, VEGF, and PD-L1, and increased the expression of caspase 3 compared to the untreated control, while histopathological changes were greater in the LV-shControl group than in the CPSF6 LV-shRNA-treated Hep3B group (Figure 8G).

## Discussion

In this study, the oncogenic mechanism of CPSF6 was explored in HCC tissues, cell lines, and xenograft and orthotopic tumor models. The TCGA database revealed that the mRNA overexpression of CPSF6 in HCC patients predicts a poor overall survival rate compared to normal controls, and that CPSF6 was overexpressed in stages I-VI of HCC. Consistently, CPSF6C is overexpressed in HCC patient tissues compared to adjacent non-cancerous tissues in a human tissue array, implying the oncogenic potential of CPSF6.

CPSF6 depletion suppressed the viability and number of colonies, induced apoptosis via cleaved-PARP, and increased the sub-G1 population in Hep3B and Huh7 cells, indicating the cytotoxic and apoptotic effect of CPSF6 depletion in HCCs; this was supported by Tan *et al.* 29 that CPSF6 is critically involved in HCC progression by upregulation of NAD(P)H quinone dehydrogenase 1(NQO1).

Recent evidence revealed that *NQO1* is negatively associated with the well-known oncogene c-Myc by decreasing Nrf2 stability 39. Thus, to explore the oncogenic mechanism of CPSF6, we elucidated the association between CPSF6 and c-Myc in HCCs. CPSF6 is endogenously expressed in parallel with c-Myc in HepG2, Hep3B, Huh7, and Sk-Hep1 cells. Consistently, CPSF6 was found to enhance the stability of c-Myc through nuclear co-localization by cycloheximide assay and immunofluorescence, which was confirmed by its binding mainly in the nucleoplasm by immunoprecipitation. Notably, the HA-c-Myc domain (258-360) directly bound to CPSF6 in Hek-293T cells cotransfected with CPSF6 and c-Myc three-domain plasmids, strongly demonstrating the binding site of c-Myc to CPSF6.

Myc levels are regulated in normal cells via targeted degradation by the ubiquitin-proteasome system (UPS) including FBW7 or SKP2 40. CPSF6 depletion promoted c-Myc degradation in Hep3B cells, which was disturbed by FBW7 depletion or the proteasomal inhibitor MG132 but not by SKP2, strongly indicating a close interaction between CPSF6 and c-Myc or FBW7. Furthermore, CPSF6 depletion attenuated the expression of c-Myc, whereas c-Myc depletion did not affect CPSF6, similar to HK2, LDH, PD-L1, and VEGF in Hep3B cells, indicating that CPSF6 acts upstream of c-Myc or HK2, LDH, PD-L1, and VEGF.

Emerging evidence indicates that the TME can be a therapeutic target for effective cancer therapy as it is modulated by components related to tumor initiation, metastasis, immune response, metabolism, and angiogenesis 41-43. Since c-Myc is involved in glycolysis 44 and cancer metabolism 45, the effect of CPSF6 on the metabolism of Hep3B cells was examined. Based on microarray data on the effect of CPSF6 on cancer metabolism and angiogenesis in CPSF6 depleted Hep3B cells, CPSF6 depletion suppressed glucose uptake, lactate and ATP, and lactate by ELISA and also attenuated the protein expression of HK2, PKM2, and LDH in Hep3B cells, indicating the potent role of CPSF6 in cancer metabolism and glycolysis, which is associated with the Warburg effect 46. Interestingly, the Warburg effect confers immune evasion to cancer cells against macrophage-mediated phagocytosis 12 and induces angiogenesis in endothelial cells in the TME 47.

Accumulating evidence reveals that tumor cells secrete pro-angiogenic factors including VEGF and bFGF to form an abnormal vascular network leading to aggressive tumor progression 48, 49 through the crosstalk between angiogenesis and immune regulation in the TME 49. As expected, CPSF6 depletion significantly reduced VEGF production and VEGF promoter luciferase activity compared to the untreated control in Hep3B cells. Furthermore, CPSF6 depletion significantly reduced the number of tube-formed networks in HUVECs compared to that in the untreated control by tube formation and CAM assays, implying the angiogenic properties of CPSF6 as a fusion partner with FGFR1.

Cancer cells can often escape from immune surveillance in the TME to inhibit the cytotoxic function of tumor-antagonizing immune cells including CD8^+^ cytotoxic T cells and effector CD4^+^ T cells via EGFR/PD-L1 signaling 50, 51. Notably, CPSF6 depletion attenuated the expression of PD-L1 and increased the percentage of CD4/CD8 cells in the splenocytes of BALB/c nude mice bearing Hep3B cells, implying an important role of CPSF6 in immune evasion.

Furthermore, the combination of Sorafenib and CPSF6 depletion significantly enhanced cytotoxicity and the G1 population and suppressed the expression of c-Myc, pro-PARP and pro-caspase3 compared to sorafenib alone in Hep3B cells, strongly demonstrating the synergistic potential of CPSF6 depletion with sorafenib for liver cancer therapy. However, further animal studies using combination therapy with Sorafenib and CPSF6 selective target compound or agents are required.

In animal studies to validate the *in vitro* effect of CPSF6, CPSF6 depletion suppressed the volume of Hep3B cells and liver weight in BALB/c mice in orthotopic and xenograft tumor models, attenuated the expression of c-Myc, PCNA, VEGF, PD-L1, HK2, PKM2, and LDH, and activated the expression of caspase 3 by immunohistochemistry, strongly demonstrating that CPSF6 can be a therapeutic target in liver cancer therapy.

**Figure 9:**
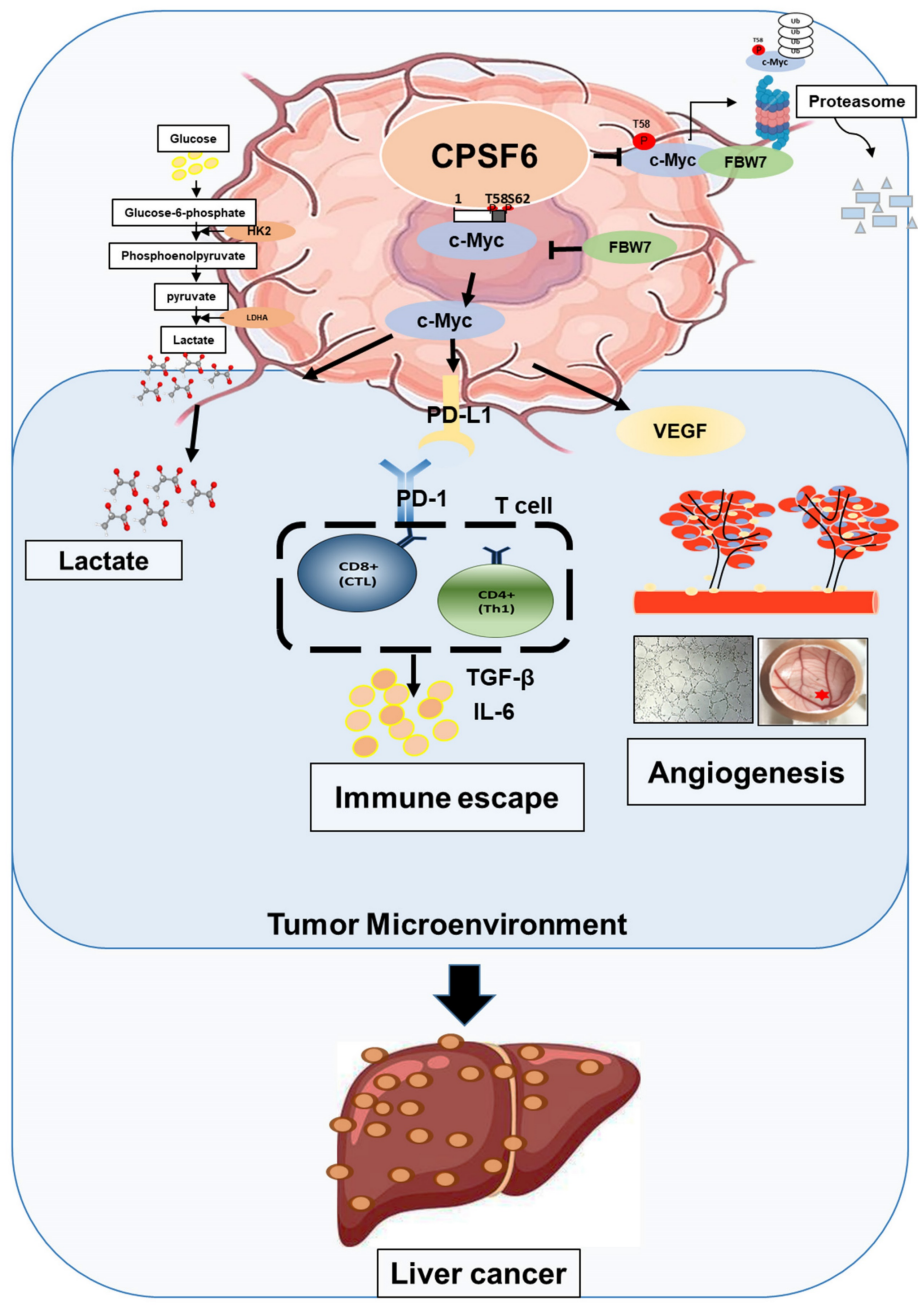
Schematic diagram on the oncogenic networks of CPSF6 via warburg effect, immune escape, and angiogenesis.

In summary, these findings suggest that CPSF6 enhances the Warburg effect and angiogenesis, leading to cancer progression via c-Myc mediated by the HK, PD-L1, and VEGF networks, with its siRNA synergistic effect with sorafenib as a molecular target in liver cancer therapy (Figure 9).

## Conclusions

These findings suggest that CPSF6 enhances the Warburg effect and angiogenesis, leading to cancer progression via c-Myc mediated by the FBW7 signaling axis, with its siRNA synergistic effect with sorafenib as a molecular target for liver cancer therapy.

## Supplementary Material

Supplementary figures.

## Abbreviations

CPSF6: Cleavage and Polyadenylation Specific Factor 6
MTT: 3-(4,5-dimethylthiazol-2-yl)-2,5-diphenyltetrazolium bromide
PARP: Poly (ADP-ribose) polymerase
ELISA: enzyme-linked immunosorbent assay
FBS: fatal bovine serum
FBW7: F-box and WD repeat domain-containing 7
HCC: hepatocellular carcinoma
HUVEC: Human Umbilical Vein Endothelial Cell
IHC: immunohistochemistry
TCGA: The Cancer Genome Atlas
TME: tumor microenvironment
PD-L1: Programmed death-ligand 1
PPI: protein-protein interactions
SKP2: S-phase kinase-associated protein 2
UPS: ubiquitin proteasome system
VEGF: vascular endothelial growth factor
bFGF: basic endothelial growth factor
CAM: Chick chorioallantoic membrane
HK2: hexokinase-2
PKM2: pyruvate kinase muscle isozyme 2
LDH: lactate dehydrogenase
PCNA: Proliferating cell nuclear antigen
