# Supplementary material for: Negative Regulation of CPSF6 Suppresses the Warburg Effect and Angiogenesis Leading to Tumor Progression Via c-Myc Signaling Network: Potential Therapeutic Target for Liver Cancer Therapy
Source: Int J Biol Sci. 2024 Jun 17;20(9):3442–60. doi: 10.7150/ijbs.93462 (PMC11234225; doi:10.7150/ijbs.93462)

Supplementary Figure 1

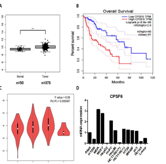

Supplementary Figure 2

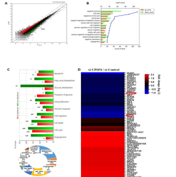

Supplementary Figure 3

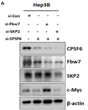

Supplementary Figure 4

A

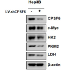

B

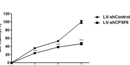

Supplementary Figure 5

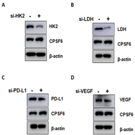

Supplement: Supplementary file 1 — Supplementary figures. [file ijbsv20p3442s1.zip › suppkementary figure.pdf]
