# Supplementary material for: Negative Regulation of CPSF6 Suppresses the Warburg Effect and Angiogenesis Leading to Tumor Progression Via c-Myc Signaling Network: Potential Therapeutic Target for Liver Cancer Therapy
Source: Int J Biol Sci. 2024 Jun 17;20(9):3442–60. doi: 10.7150/ijbs.93462 (PMC11234225; doi:10.7150/ijbs.93462)
Supplement: Supplementary file 1 — Supplementary figures. [file ijbsv20p3442s1.zip › Supplementary Figure Legends.docx]

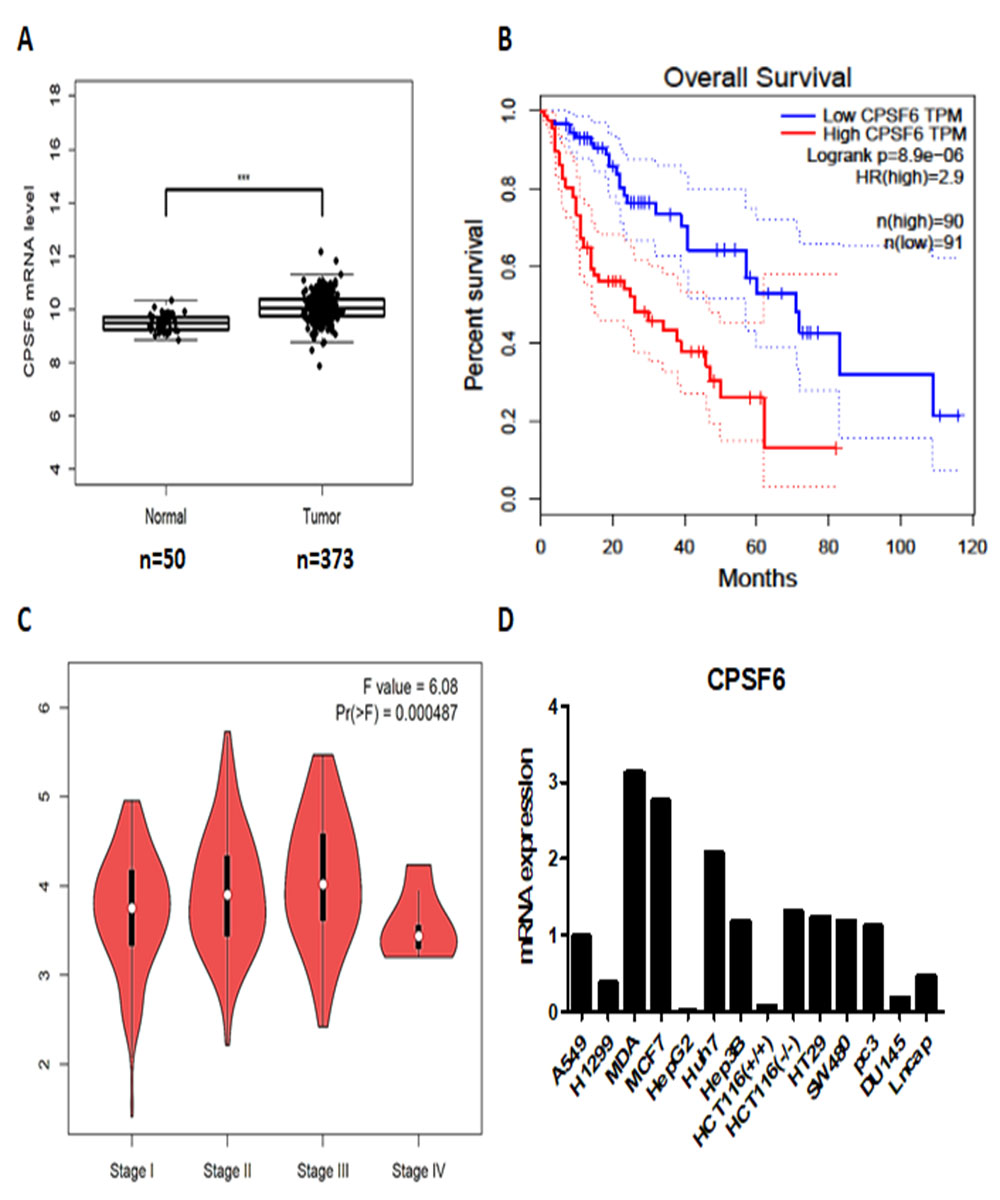


**Figure S1. CPSF6 expression was overexpressed in HCC patient tissues and cancer cells.** (A) CPSF6 mRNA level in tissues of normal and HCC patients by TCGA analysis (B) Effect of CPSF6 on the survival rate in the patients with HCC. (C) Relationship between CPSF6 and the stages of HCC. (D) Endogenous mRNA expression levels of CPSF6 in various cancer cell lines.


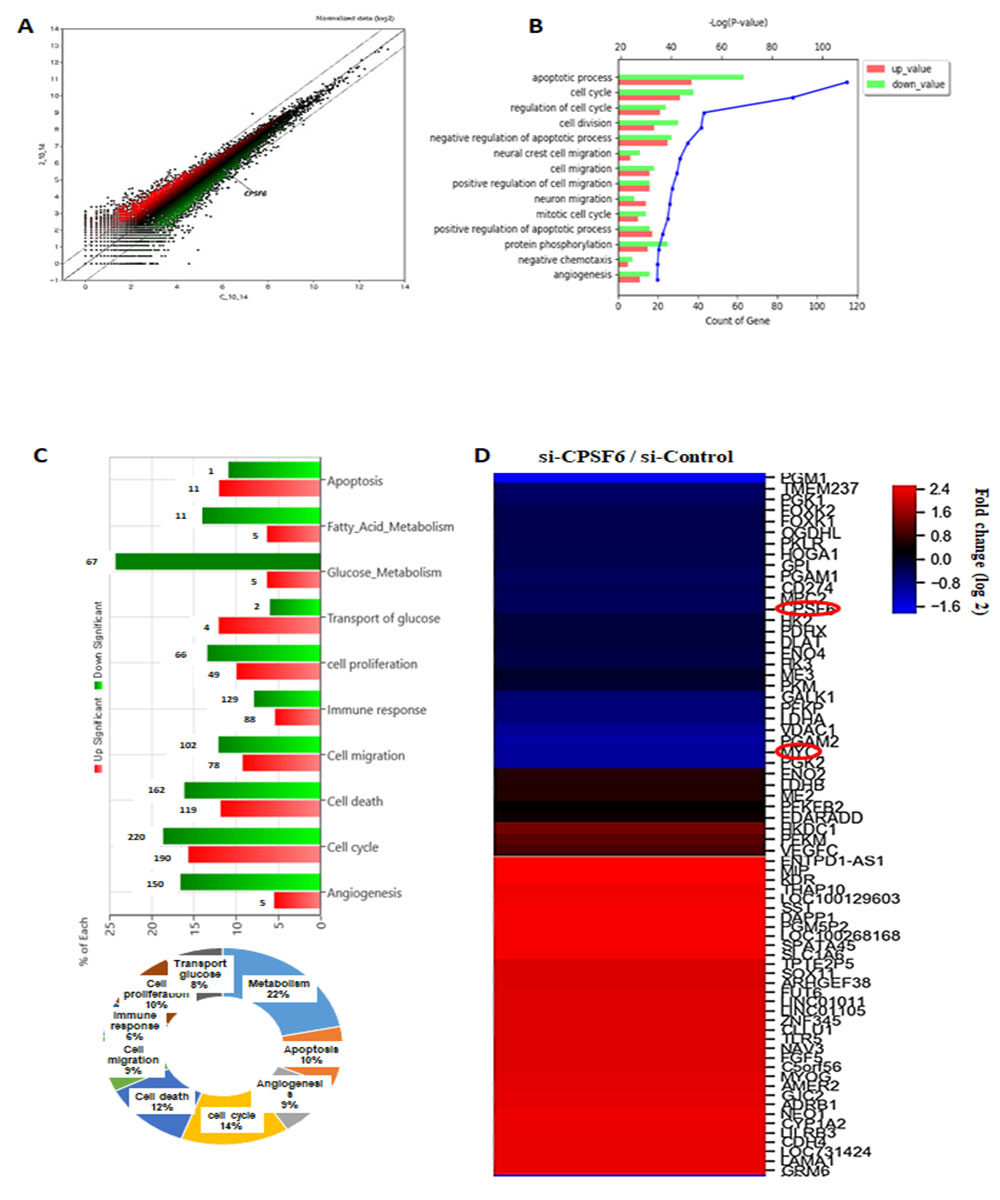


**Figure S2. Differentially expressed gene profile and signaling pathways in CPSF6 depleted Hep3B cells.** (A) Upregulated (Red) and downregulated(Blue) genes in CPSF6 depleted Hep3B cells compared to untreated control by Scatter plot. (B) Associated signaling pathways in CPSF6 depleted Hep3B cells compared to untreated control by GO analysis. (C) Repressed signaling pathways (Green) and induced signaling pathways (Red) in CPSF6 depleted Hep3B cells by KEGG analysis. (D) Upregulated and downregulated genes in CPSF6 depleted Hep3B cells by mRNA sequence based analysis. Red and blue colors in heat map indicate increased and decreased expression levels, respectively.


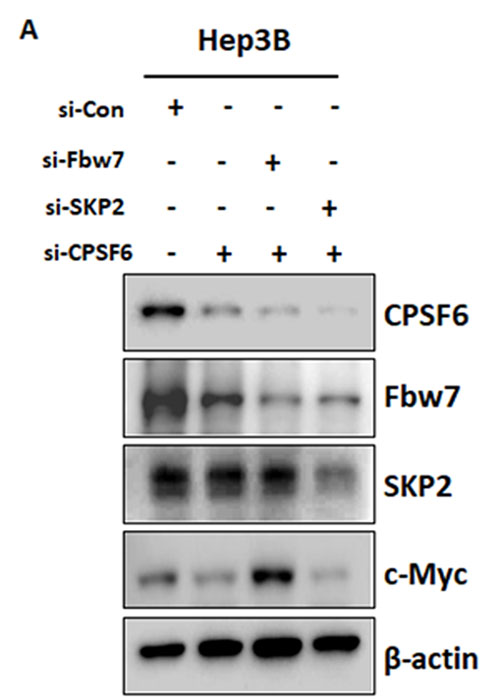


**Figure S3. FBW7 depletion restored the expression of c-Myc targeted by CPSF6 depletion in HCC.** Effect of FBW7 or SKP2 depletion on c-Myc expression in Hep3B cells. Hep3B cells transfected with control or CPSF6 siRNA plasmid were co-transfected with FBW7 or SKP2 siRNA plasmid and also were subjected to Western blotting.


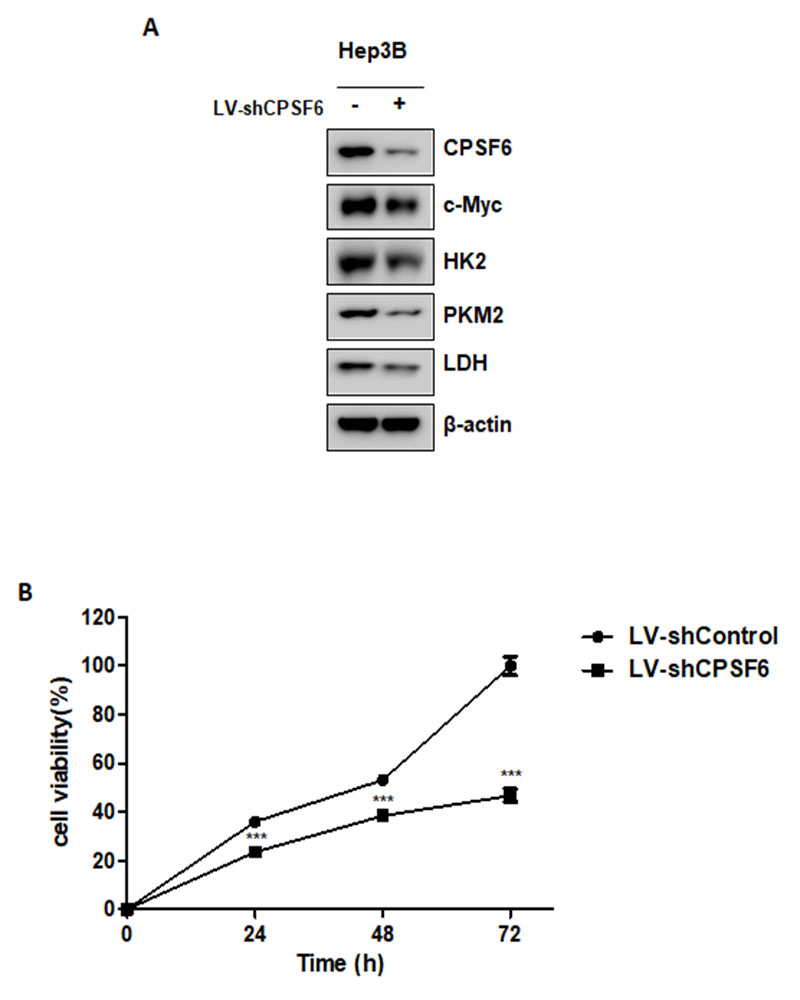


**Figure S4.** **Establishment of CPSF6 shRNA suppressed proliferation and related genes in HCC** (A) Inhibitory effect of LV-CPSF6 on CPSF6, c-Myc, HK2, PKM2, LDH in Hep3B cells. (B) Inhibitory effect of LV-CPSF6 on the viability of Hep3B cells. ***p<0.001 vs untreated control.


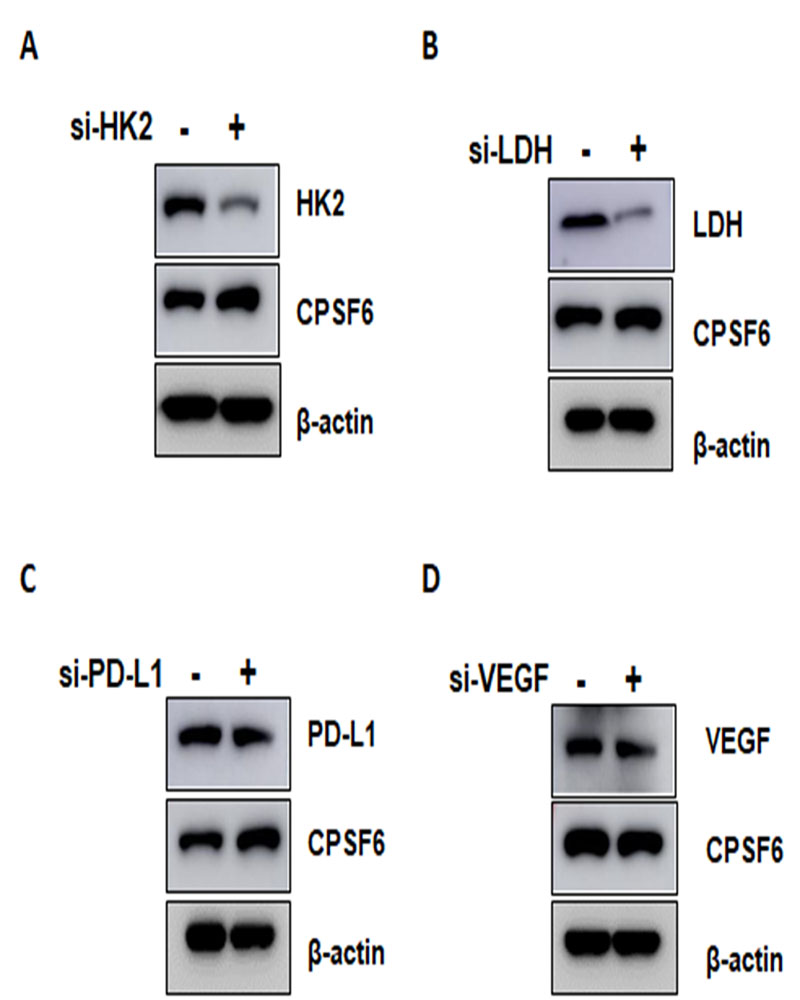


**Figure S5.** **Depletion of HK2, LDH, PD-L1 and VEGF did not affect CPSF6 in HCCs. (A)** Effect of HK2 on CPSF6 in Hep3B cells. **(B)** Effect of LDH on CPSF6 in Hep3B cells. **(C)** Effect of PD-L1 on CPSF6 in Hep3B cells. **(D)** Effect of VEGF on CPSF6 in Hep3B cells.
